# Feasibility and outcome of primary laparoscopic cytoreductive surgery for advanced epithelial ovarian cancer: a comparison to laparotomic surgery in retrospective cohorts

**DOI:** 10.18632/oncotarget.22573

**Published:** 2017-11-03

**Authors:** Huamao Liang, Hongyan Guo, Chunyu Zhang, FuLi Zhu, Yu Wu, Kun Zhang, Hua Li, Jinsong Han

**Affiliations:** ^1^ Department of Obstetrics and Gynecology, Peking University Third Hospital, Beijing, P.R. China

**Keywords:** ovarian cancer, epithelial, laparoscopy, cytoreductive surgery, primary

## Abstract

**Objectives:**

To assess the feasibility and outcome of primary laparoscopic cytoreductive surgery on advanced epithelial ovarian cancer in comparison with conventional open surgery.

**Materials and Methods:**

Patients undergoing primary laparoscopic cytoreductive surgery (LCS) from March 2007 to December 2016 were matched to controls treated with laparotomic cytoreduction during the same period. Procedural data and outcomes were analyzed.

**Results:**

The LCS group (*n* = 64) and laparotomic group (*n* = 68) had similar age, BMI, stages, histologic type and grading. The LCS group exhibited significantly less operating time (*P* < 0.001), less intraoperative blood loss (*P* < 0.001), and shorter time to recover postoperatively (*P* = 0.002). No statistical difference was observed for the number of pelvic and para-aortic lymph nodes dissected (*P* = 0.326 and *P* = 0.151). Significant difference was observed in satisfaction of the cytoreduction (95.3% vs. 76.5%, *P* = 0.008). No significant difference were observed either in intra-operative or in post-operative complications between the two groups (*P* = 0.250). Three patients in the LCS group experienced intra-operative complications (4.7%) and were all treated laparoscopically. The conversion rate was 3.1%. No significant differences were observed in the progression-free survival and overall survival between the two groups during the medium follow-up of 18 months (*P* = 0.236 and *P* = 0.216). The 2-year and 3-year progression-free survival was 67.9%, 55.5% in LCS group and 53.8%, 33.3% respectively in the control group. The 2-year and 3-year overall survival was 95.8%, 88.7% respectively in the LCS group and 89.0%, 83.7% in the control group.

**Conclusions:**

Primary laparoscopic cytoreductive surgery in some strictly selected advanced stages of EOC patients was feasible and safe, resulting in oncologic outcomes not inferior to those in open surgery.

## INTRODUCTION

Ovarian cancer has the highest mortality in all the gynecological malignancies. Epithelial ovarian cancer (EOC) is the most common and lethal subtype. Approximately two thirds of EOC cases are detected at stage III or IV because of the difficulties in early diagnosis. Cytoreductive surgery is the most common primary treatment for these patients and has been performed by open surgery conventionally. Although laparoscopy has been shown to be safe and effective in treating patients with early stage EOC, few surgeons have adopted this approach to treat patients with the advanced disease [1]. The complexities of advanced stage EOC, such as local tissue invasion and extensive metastases in the abdominal cavity, significantly increases the difficulty of laparoscopic cytoreductive surgery (LCS). Procedural challenges include separation of local adhesions, treatment of massive bleeding when it occurs, resection of the omentum heavily burdened by metastases, and resection of metastatic lesions on the diaphragm or other difficult sites. These challenges have hindered the adoption of laparoscopic techniques, and so far, only limited data on primary laparoscopic cytoreduction for advance stage EOC have been published [2–4]. In order to overcome the above problems, some randomized controlled trial studies have demonstrated that patients with optimal cytoreduction after neoadjuvant chemotherapy have approximately the same survival rate as patients optimally cytoreduced at primary debulking surgery [5]. However, It is an fact that neoadjuvant chemotherapy may lead to chemoresistance, which results in relapses, affects the survival and subsequent treatment of the patients [6]. Primary debulking surgery with optimal cytoreduction followed by platinum based chemotherapy is still the current first line treatment in patients with advanced ovarian cancer. Here we present our experience of 64 cases of primary LCS from 2007 to 2016, in comparison with patients treated with laparotomic cytoreduction. The primary objective of this study was to assess feasibility of primary laparoscopic cytoreduction in patients with EOC. Secondary objectives were assessing perioperative outcomes and survival.

## RESULTS

From March 2007 to December 2016, ninety-five patients received laparoscopy and were diagnosed as EOC with advanced stages. Sixty-four patients among them received primary laparoscopic cytoreduction directly following the laparoscopy. Eighteen patients received neoadjuvant chemotherapy afterword. Thirteen patients were converted to primary laparotomic cytoreduction immediately according to surgeons’ experience or general surgeons’ opinion, these patients and other 55 patients with advanced stage of EOC (diagnosed according to pre-operative examination, CT scans and laparotomy) who were treated by open surgery were chosen to be controls.

Detailed clinical character, tumor staging or histology are summarized in Table 1. Most of the patients were classified as stage III, especially stage IIIc, with bulky metastases ≥ 2 cm. No significant difference could be seen in the clinical characteristics between the two groups. Table 2 presents intra- and postoperative details. The mean operating time (304.5 ± 111.7 min vs. 389.0 ± 117.4 min) and mean blood loss (232.2 ± 295.0 ml vs. 769.6 ± 613.2 ml) were significantly lower in the LCS group than in the control group (*P* < 0.001). In the LCS group, 6 patients (9.4%) required intraoperative blood transfusion (400 ml and 2000 ml), whereas in the control group, 38 patients (55.9%) required blood transfusion (400 ml to 3200 ml). One patient in LCS group and 11 patients in control group haven't received systematic lymphadenectomy. For the other patients, no statistical difference was observed for the mean number of pelvic (15.6 ± 7.9 vs. 17.0 ± 8.4), or para-aortic lymph nodes (5.7 ± 5.0 vs. 4.6 ± 3.7) dissected (*P* = 0.326 and *P* = 0.151).

**Table 1:** Clinical characteristics of epithelial ovarian carcinoma (EOC) treated with laparoscopic vs. laparotomic cytoreductive surgery

Variables	Laparoscopic surgery	Laparotomic surgery	*P* value
Number of patients	64	68	
Age ( years, mean ± SD)	53.5 ± 11.4	51.9 ± 11.8	0.43
Gravida [mean ± SD]	2.6 ± 1.3	2.8 ± 1.7	0.45
Para [mean ± SD]	1.6 ± 1.5	1.5 ± 1.0	0.66
Body Mass Index (kg/m^2^, mean ± SD)	24.1 ± 4.6	23.0 ± 3.6	0.13
FIGO Stages			0.19
Stage II, *n* (%)	17 (26.6%)	11 (16.2%)	
IIa	7	4	
IIb	9	7	
Stage III, *n* (%)	43 (67.2%)	48 (70.6%)	
IIIa1	4	4	
IIIa2	4	3	
IIIb	9	6	
IIIc	26	35	
Stage IV	4 (6.25%)	9 (13.2%)	
Histopathology *n*(%)			0.21^a^
Serous	53 (82.8%)	60 (88.2%)	
Mucinous	0 (0.0%)	2 (2.9%)	
Clear cell	7 (10.9%)	2 (2.9%)	
Endometroid	2 (3.1%)	3 (4.4%)	
Malignant Brenner tumor	1 (1.6%)	0 (0.0%)	
Mixed	1 (1.6%)	1 (1.5%)	
Grading *n*(%)			0.74
Low grade	9 (14.0%)	11 (16.2%)	
High grade	55 (86.0%)	57 (83.8%)	

SD, standard deviation, ^a^Fisher's exact test

**Table 2:** Procedural data in laparoscopic vs. laparotomic cytoreductive surgery groups

Variables	Laparoscopic surgery	Laparotomic surgery	*P* value
Number of patients	64	68	
Largest tumor diameter (cm, mean ± SD)	7.9 ± 4.6	10.8 ± 5.2	0.001
Operative time (min, mean ± SD)	304.5 ± 111.7	389.0 ± 117.4	< 0.001
Estimated blood loss (ml, mean ± SD)	232.2 ± 295.0	769.6 ± 613.2	< 0.001
Numbers of pelvic lymph nodes (*n*, mean ± SD)	15.6 ± 7.9	17.0 ± 8.4	0.326
Numbers of para-aortic lymph nodes (*n*, mean ± SD)	5.7 ± 5.0	4.6 ± 3.7	0.151
Satisfaction of the surgery, *n*(%)			0.008
Sub-Optimal	3 (4.7%)	16 (23.5%)	
Optimal (No residul nodules)	55 (85.9%)	46 (67.6%)	
Optimal (Residul nodules) ≤ 1 cm	6 (9.4%)	6 (8.8%)	
Complications, *n* (%)			0.250^a^
Intra-operation	3 (4.7%)	4 (5.9%)	
Post-operation( ≤ 1 month)	5 (7.8%)	11 (16.2%)	
Conversion to open surgery	2 (4.2%)	0 (0.0%)	
Average time to bowel movement (days, mean ± SD)	1.7 ± 0.8	2.7 ± 1.1	0.002
Time to first cycle of adjuvant chemotherapy (days, mean ± SD)	10.8 ± 6.9	20.0 ± 18.6	< 0.001

SD, standard deviation, ^a^ Fisher's exact test

Significant differences were observed in achievement of satisfaction of the cytoreduction between the two groups (*P* = 0.008). 95.3% (61/64) of the patients in LCS group and 76.5% (52/68) of the patients in control group achieved optimal cytoreduction, with no residual tumor 85.9% (55/64) in the LCS group and no residual tumor 67.6% (46/68) in control group.

In the LCS group, six patients experienced rectosigmoid resection and one experienced small bowel resection to achieve optimal cytoreduction, while in the control group, 16 rectosigmoid resection, two small bowel resection and two splectomy were performed to achieve the same goal.

4.7% patient in LCS group and 23.5% of the patients in the control group could not achieve optimal cytoreduction, with the residual tumors located in liver, diagram, huge lymph nodes around the Inferior vena cava or above the level of the renal vein, or unresectable flattened masses along the base of the small-bowel mesentery. Refusal by the patients or the relatives to have bowel resection and colostomy when multiple segments of intestine were involved was also a reason for sub-optimal cytoreduction.

No significant differences were observed either in intra-operative or in post-operative complications between the two groups (*P* = 0.250). In the LCS group, three patients experienced intraoperative complications (ureter injury, external iliac vein injury, and Obturator nerve injury respectively). The ureteral injury was repaired laparoscopically and ureteral stent was installed. The external iliac vein injury and the Obturator nerve injury were sutured with 5-0 and 3-0 absorbable suture laparoscopically respectively. Postoperatively, in LCS group, one case of hydronephrosis, two cases of ileus, one case of thrombosis of lower limb and one case of pneumonia occurred. These complication were resolved after conservative management. In the control group, one bladder injury, one pancreas injury and one external iliac artery injury and one Inferior vena cava injury occurred intraoperatively. All patients recovered after intra-operative repair and drainage. Postoperatively, two patients suffered from poor abdominal wound healing, five patients suffered from thrombosis of lower limb (including two developing pulmonary emblolism), three patients suffered from ileus and one suffered from lower gastrointestinal hemorrhage. These complications were again managed medically.

Two cases (3.1%) of conversion to open laparotomy occurred in LCS group. One patient was converted due to severe pelvic adhesion. After laparoscopic para-aortic lymphadenectomy, omentectomy and partial pelvic lymphadenectomy, laparotomic procedures were performed to complete hysterectomy and bilateral salpingo-oophorectomy and partial pelvic lymphadenectomy. The other patient converted to open surgery to perform omentectomy because of the huge mass and severe adhesion of the omentum.

The average time to bowel movement was significantly shorter in the LCS group (1.7 ± 0.8 days vs. 2.7 ± 1.1 days, *P* = 0.002). The average time from cytoreduction to the first cycle of adjuvant chemotherapy was also shorter in the LCS group (10.8 ± 6.9 days vs. 20.0 ± 18.6 days, *P* = 0.002).

All patients were treated with 6-8 cycles of taxol (intravenous) and carboplatin or cisplatin (intravenous or intraperitoneal) as adjuvant chemotherapy.

**Figure 1:**
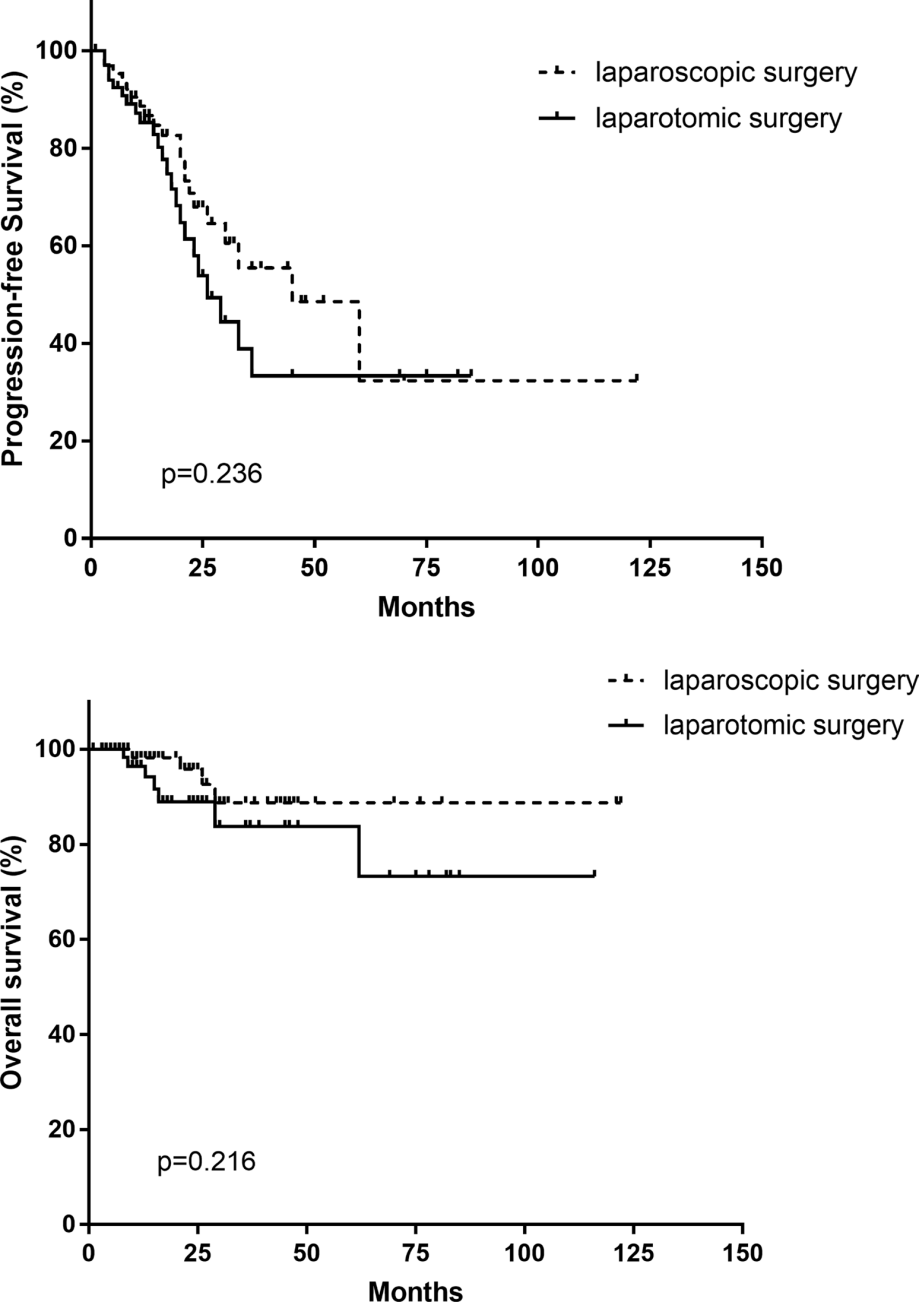
Progression-free survival and overall survival in laparoscopic vs. laparotomiccytoreductive surgery groups

All the patients were followed up 5–122 months, with the medium follow-up period as 18 months. No significant differences were observed in the progression-free survival and overall survival between the two groups (*P* = 0.236 and *P* = 0.216, Figure 1). The 2-year and 3-year progression-free survival was 67.9%, 55.5% in LCS group and 53.8%, 33.3% respectively in the control group. The 2-year and 3-year overall survival was 95.8%, 88.7% respectively in the LCS group and 89.0%, 83.7% in the control group.

## DISCUSSION

### Current role of laparoscopy in advanced ovarian cancer

Since 2006, laparoscopy has been introduced in advanced stage of EOC management to identify patients deemed unresectable at primary surgery, obtaining a histological diagnosis, assessing resectability and avoiding a large explorative laparotomy [7, 8]. Limited data showed that gynecologic oncologists were inclined to perform interval laparoscopic cytoreduction because of the reduced difficulty of the surgery followed neoadjuvant chemotherapy [5, 9, 10]. So far, there have been very limited data on primary laparoscopic cytoreductive surgery for advanced EOC [2–4].

Nezhat [2] firstly reported 13 cases of primary cytoreduction, with 76.9% patients reached optimal cytoreduction. In their another report [3], 88.2% of 17 patients reached optimal cytoreduction. Patients undergoing laparoscopy had less blood loss and a shorter hospital stay compared with the laparotomy group, no significant complications was seen. A potentially prolonged recurrence interval in the laparoscopic group was seen in their study. Fanning [4] also published a study of 25 patients who underwent primary laparoscopic debulking and reported a cytoreduction rate of 92%, in which 36% had no residual diseases. Two procedures were converted to laparotomy because of extensive omental metastasis and bulky metastasis surrounding the rectosigmoid. Post-operative complications rate was 24%, and median overall survival was 3.5 years.

In this paper, we reported a series of 64 cases of primary laparoscopyic cytoreduction, with 68 primary laparotomic cytoreduction as control. We found that the LCS group had a significantly shorter operation time and less blood loss than the open cytoreductive surgery group. The intra-operative and post-operative complication rates were comparable. The conversion rate of LCS group was 3.1%. Laparoscopic surgery also offers faster postoperative recovery. The 2-year and 3-year progression-free survival was slightly higher in LCS group (67.9%, 55.5%) than in the control group (53.8%, 33.3%). However, No significant differences were observed either in progression-free survival or overall survival between the two groups.

### Advantages of laparoscopic surgery for cytoreduction

Consistent with the report by Nezhat [3], our study showed that blood loss was significantly reduced in laparoscopic cytoreduction compared to open surgery. Rapid recovery of the patients, and significantly shorter period to initiation of adjuvant chemotherapy were also be showed in our study and other reports [2–4].

Another advantage of the laparoscopic approach is lymphadenectomy. In laparoscopy, the region around the abdominal aorta and the Inferior vena cava is more visible and reachable, which facilitates the local lymph node dissection. The number of lymph nodes dissected during cytoreduction is often an indicator of the thoroughness of the treatment. The results of this study show that laparoscopic surgery can achieve the same thoroughness as the open surgery, with a similar number of lymph nodes dissected; these results are also consistent with the literature [10].

### Challenges of primary laparoscopic surgery for cytoreduction

The complexities of advanced stage EOC, such as local tissue invasion and extensive metastases in the abdominal cavity, significantly increase the difficulty of primary laparoscopic cytoreductive surgery. Procedural challenges include separation of local extensive adhesions and invasion, treatment of massive bleeding, resection of the omentum heavily burdened by metastases, and resection of metastatic lesions on the diaphragm or other difficult sites. These challenges have hindered the adoption of laparoscopic techniques, and so far, only limited data on primary laparoscopic cytoreduction for advance stage EOC have been published [2–4]. Here we list some of the strategy we conformed in our LCS surgery.

First: control of blood loss in a LCS surgery. The separation and resection of the primary ovarian lesion may couple with massive bleeding, which sometimes can account for more than 50% of the total blood lost. To reduce local hemorrhage, our strategy is to initially separate and electrocoagulate the ovarian artery and vein. Then the retroperitoneum is entered laterally. The two layers of the broad ligament were incised, the mass was seperated retroperitoneally. The retroperitoneal space is usually looser and easier to separate. If the local peritoneum is obviously involved, it can also be separated from the retroperitoneal space at the same time. Then ipsilateral utero-ovarian ligament and fallopian tube were electrocoagulated and incised. Thus the ovarian mass, the surrounding adhesion and the metastasis were separated using the retroperitoneal approach with less blood lost. Small dry gauze put in through the 10 mm Trocar could be an effective hemostatic method for patients with diffuse blood oozing and unclear visual field. Alternatively, under these circumstances, surgeons could firstly perform those procedures which are easier to do, like omentectomy, lymphanectomy, and leave the most difficult part to be treated in the end. Adjustment of the order of procedures give us more possibility to complete the laparoscopic cytoreduction with clearer vision and less stress.

Second: how to treat the bulky tumor mass of omentum. When the omentum contains extensive metastasis, omentectomy could be difficult. If the omental “cake” was observed to be densely adherent to the transverse colon and complete resection is impossible, further procedures should be aborted, and neoadjuvant chemotherapy followed by interval cytoreductive surgery is recommended. If omentum is explored to having the possibility to be removed by laparoscopy, the omentum must be carefully pulled in the cephalad, the omentum should be resected along the serosa of the transverse colon and the greater curvature of the stomach by harmonic scalpel. Attention should be paid to prevent from tearing the hilum of spleen or gastro-omental artery, which can result in massive bleeding. We recommend a 5th trocar (5 mm) in the right upper quadrant as it can be useful when resecting the omentum.

Third: issues about para-aortic lymphadenectomy. We performed systematic lymphadenectomy in advanced EOC patients, because systematic lymphadenectomy might have therapeutic value and improve prognosis for patients with optimally cytoreduced advanced ovarian cancer [11]. For those stage IIIc patients who cannot gain optimal cytoreduction, lymphadenectomy was not entirely necessary. The challenge in para-aortic lymphadenectomy is how to remove the local lymph nodes without damaging the surrounding tissues, such as aorta, inferior vena cava, ureteral, duodenal, renal vein and inferior mesenteric artery. Rupture of the inferior vena cava is the most likely occurred complication in this procedure, and the result is catastrophic. It is relatively difficult to suture the vessel laparoscopically, so most of the complications in this portion need to be repaired by conversion to open surgery. Perfect exposure is the crucial step. The anterior peritoneum can be pulled up or stitched to the anterior abdominal wall. The ureter should be pushed away from the inferior vena cava. After the full exposure of surounded organs, en bloc resection could be performed gentlely and gradually with harmonic scalpel. Similarly, if local lymph nodes are massive, fused and fixed, the para-aortic lymphadenectomy should be given up, and neoadjuvant chemotherapy followed by interval cytoreductive surgery is recommended.

Fourth: how to achieve optimal debulking. For metastases disseminated in the pelvis, on the abdominal peritoneum, the intestinal mesentery, or diaphragm, vacuum aspiration and bipolar coagulation could be a good alternative if Argon Beam Coagulator or Cavitron ultrasonic surgical aspirator (CUSA) is not available. Resection of metastases in the mesentery should also be carefully performed so as not to affect the intestinal blood supply or cause subsequent intestinal necrosis. Large metastases on the intestinal surface should be careful evaluated. Seromuscular metastases could be removed by using a harmonic scalpel, with the lesions stitched with absorbable sutures. For full-thickness bowel metastases and other organ metastases, we recommend obtaining assistance from general surgeons to complete the operation either via laparoscopy or laparotomy, according to their choice.

Fifth: the port-site metastasis. This is another concerning complication of the laparoscopic surgery, with a reported incidence of 1.18% to 16% [12]. No port-site metastasis occurred in our study during a median follow-up of 18 months; however, given the relative short follow-up, this risk needs to be further assessed in longer follow-up.

Sixth: how to avoid and treat complications. In advanced stages of EOC, the metastatic condition is often complicated. Because of the large amount of ascites, the abdominal organs and the peritoneum are often edematous and fragile. Intraoperative peritoneal effusion and bleeding may compromise the visual field. Therefore, the surgeon should be very careful in every procedure. Surgeons must have excellent experience in open cytoreductive surgery, be extensively skilled in laparoscopic staging surgery, and can deal with most of the complications occurred in laparoscopic procedures. In laparoscopic surgeries, organ injury is the main reason of conversion to open surgery. The conversion rate of laparoscopic staging surgery of EOC was reported to be 4%–10% [13], or 6.6% in laparoscopic cytoreduction [14]. In our study, the conversion rate was 3.1%. All the intra-operative complications (ureter injury, external iliac vein injury, and Obturator nerve injury respectively) were repaired laparoscopically. In brief, laparoscopic cytoreductive surgery for advanced ovarian carcinoma in strictly selected patient is relatively safe, as long as the surgeon can avoid major injuries and is capable to treat intra-operative complications with appropriate suture techniques for the bowel, ureteral or the vascular injuries. Moreover, it is of great importance to select the patients who are really suitable to receive the surgery and get benefit from LCS.

### Patients’ selection and the bias of the study

In our study, although no significant differences could be seen in the clinical characters between the two groups, more stage II, stage IIIa and IIIb, but less stage IIIc and stage IV patients were seen in the LCS group (*P* = 0.19). Besides, the largest tumor diameter in LCS group is significantly smaller than that in laparotomy group (7.9 ± 4.6cm vs. 10.8 ± 5.2 cm, *P* = 0.001). This is the result of our two steps strictly selection criteria. Firstly, in the pre-operative evaluation, surgeons were inclined to leave those patients who had large masses with questionable resectability to the group of open surgery. Then the investigative laparocopy re-evaluated the abdomimal metastatic status, and excluded those patients who could not be resected easily by laparoscopic procedure and chose those patients who has less metastasis, with less adhesion and invasion to receive laparoscopic cytoreduction. Here, we refer to PIV score systerm published by Fagotti et al [8]. It is clear that in open surgery group, the following conditions are more common: large tumors involving in the bowel which need to be treated by enterectomy and entero-anastomosis, or colostomy; the omentum cake which is difficult to separate and resected, splenectomy or other more procedures need to be performed by general surgeons. These strict selection may account for the result in our study, that more optimal cytoreduction rate could be reached in LCS group than that in the control group (95.3% vs. 76.5%, *P* = 0.008).

Although no significant difference in progression-free survival and over-all survival were seen in our study, and the 2-year and 3-year overall survival was similar in the two groups (95.8%, 88.7% respectively in the LCS group and 89.0%, 83.7% in the control group), the 2-year and 3-year progression-free survival was slightly higher in LCS group (67.9%, 55.5%) than in the control group (53.8%, 33.3%). Even taking into account certain factors such as selection bias, our data indicated that, at least, the oncologic outcome of primary laparoscopic cytoreduction is not inferior to that in the open surgery. These results also indicate that, in advanced stage EOC patients who have been critically selected, primary laparoscopic cytoreductive surgery is feasible and safe, exhibiting compatible postoperative outcomes to those seen in the laparotomic group.

### Limitation of the study

Like the limited literature, our study is also limited by its retrospective design, and relative short follow-up. However, it is among the first that compared outcomes of primary laparoscopic vs. laparotomic cytoreduction in cohorts study with the largest number of cases till now (64 in LCS group vs. 68 in the control group). To date, laparoscopic procedure is still have not recommended for primary cytoreduction in EOC patients. Large prospective trials are needed to assess the feasibility of primary laparoscopic-assisted cytoreduction, considering the real implications on the oncologic and surgical outcome and quality of life of these patients (LE IIIB) [15].

## MATERIALS AND METHODS

### Patients

This retrospective analysis includes patients who underwent primary laparoscopic or laparotomic cytoreductive surgeries for advanced EOC from March 2007 to December 2016 at Peking University Third Hospital. After exclusion of patients with borderline tumor, multiple cancinoma (endometrial/ovary cancer, lung/ovary cancer), with neoadjuvant chemotherapy or with incomplete clinical or surgery-pathologic data, 64 patients primarily treated with LCS were submitted into the study group. Sixty-eight patients who had received primary laparotomic cytoreductive surgery at the same institution and during the same period were chosen as controls.

### Laparoscopic cytoreduction technique

Under general anesthesia, the patient was placed in the dorsolithotomy and maximal Trendelenburg position. The first trocar (10 mm) was inserted above or below the umbilicus after pneumoperitoneum was created. The entire abdominal cavity was inspected systematically under direct vision. Patients were submitted to laparoscopic cytoreduction only if they were predicted to achieve optimal debulking by the surgeon as previously reported [3]. Otherwise, only biopsies (through a 2^nd^ 5 mm trocar in the left lower quadrant) were performed, and the patient would be treated with neoadjuvant chemotherapy for 2–3 cycles before a secondary laparoscopy and interval cytoreductive surgery. Or, if bowel resection or other more complicated procedures were required to achieve an optimal cytoreduction, the patients were treated laparoscopically or converted to open surgery according to general surgeon's decision. If decision of laparoscopic cytoreductive surgery is made, the vaginal delineator was placed, then the 3^rd^ and the 4^th^ trocar (5 mm each) are inserted in the left and right lower quadrant. We recommend a 5^th^ trocar (5 mm) in the right upper quadrant as it can be useful when resecting the omentum.

Laparoscopic cytoreductive surgery consists of exploration, collection of peritoneal washing or ascites for cytologic examination, total hysterectomy, bilateral salpingo-oophorectomy, total infracolic omentectomy, pelvic and para-aortic lymphadenectomy and/or appendectomy, as well as removal of all visible tumors and cauterization of small nodules with bipolar coagulation. If optimal cytoreduction cannot be reached, pelvic and para-aortic lymphadenectomy might not be performed in some patients. When common iliac and para-aortic lymphadenectomy were performed, the laparoscope was rotated clockwise for 90 degrees, the peritoneum over the right common iliac artery was opened, and the incision was extended cephalad over the underlying inferior vena cava and abdominal aorta. Then common iliac, precaval and paraaortic nodal dissection was performed. The upper limit of the nodal dissection was below the renal vein, or at least above the level of Inferior mesenteric artery. Appendectomy was performed by coagulation of the mesoappendix, ligation of the appendix by non-absorbable sutures, and resection. Bowel resection, splenectomy or other more procedures were performed by general surgeons. Optimal cytoreduction is defined as a maximal diameter ≤ 1 cm of the largest residual tumor at the completion of the primary operation.

### Laparotomic cytoreduction technique

The patient was positioned in the lithotomy position or a supine straddle position under the general anesthesia. In all cases, laparotomy was performed via a midline longitudinal incision, with the same cytoreductive procedures as described above.

The patients were treated with antibiotics (for 2–3 days) and heparin (started 24–48h postoperatively till the patients discharged from the hospital). Clinicopathological staging was made postperatively. The patients were treated with Taxol /platin-based chemotherapy for one cycle and then discharged. All the patients received 6–8 cycles of chemotherapy postoperatively. The patients were followed up routinely after the completion of the treatment.

### Data collection

The following data were collected from the patients’ records: age, body-mass index (BMI), surgical history, clinicopathological stage, histological type and grade, operating time (from the skin incision to the end of surgical procedure), estimated blood loss, blood transfusion, surgical complications (defined as bowel, bladder, ureter or vascular injuries, or thrombosis during the surgery or within one month postoperatively), hospital stay (defined as the duration from the date of surgery till when the patient was discharged after the first chemotherapy postoperatively), progress-free survival, and overall survival. All patients were followed up till April 30, 2017, death, or loss to follow-up.

### Statistical analysis

Study data were collected on standard case report forms, checked for completeness, and double keyed into an Excel 2010 database. All analyses were conducted with SPSS version 18.0 (SPSS Inc., Chicago, IL, USA). Continuous data were described as mean ± standard deviationor median (25 percent, 75 percent), and the Student's *t*-test or non-parametrictest was used to compare differences between groups. Categorical data were described as frequencies and proportions and were compared by the Chi-square test or Fisher's exact test. The Kaplan-Meier analysis was used to analyze overall and progression-free survival and log-rank test was used to compare difference between groups. A two-sided *p* value < 0.05 was considered statistically significant in all analyses.

## CONCLUSIONS

Overall, results from this study show that laparoscopic cytoreductive surgery in some strictly selected advanced stages of EOC patients was feasible and safe, resulting in outcomes not inferior to those in open surgery. We showed that the laparoscopic approach offers advantages of less operative blood loss and shorter post-operative recovery; however, we recognize that there are still challenges and limitations in performing laparoscopic surgery. More studies are needed to further evaluate this approach in advanced-stage EOC. With accumulating techniques and experience, laparoscopic surgery may benefit more patients with advanced EOC.

## Abbreviations

EOC: epithelial ovarian carcinoma
LCS: laparoscopic cytoreductive surgery
